# Digital education for health professionals in India: a scoping review of the research

**DOI:** 10.1186/s12909-023-04552-2

**Published:** 2023-08-09

**Authors:** Lasse X Jensen, Alexandra Buhl, Suhaib Hussain, Anup Karan, Flemming Konradsen, Margaret Bearman

**Affiliations:** 1 Department of Public Health, University of Copenhagen, Øster Farimagsgade 5, Bdg. 9, Copenhagen K, 1353 Denmark; 2 Public Health Foundation of India, New Delhi, India; 3 Novo Nordisk Foundation, Copenhagen, Denmark; 4 Centre for Research in Assessment and Digital Learning, Deakin University, Melbourne, Australia

**Keywords:** Health professions education, India, Digital education, Scoping review

## Abstract

**Background:**

The World Health Organization (WHO) predicts a global shortfall of 18 million health workers by 2030, particularly in low- and middle-income countries like India. The country faces challenges such as inadequate numbers of health professionals, poor quality of personnel, and outdated teaching styles. Digital education may address some of these issues, but there is limited research on what approaches work best in the Indian context. This paper conducts a scoping review of published empirical research related to digital health professions education in India to understand strengths, weaknesses, gaps, and future research opportunities.

**Methods:**

We searched four databases using a three-element search string with terms related to digital education, health professions, and India. Data was extracted from 36 included studies that reported on empirical research into digital educational innovations in the formal health professions education system of India. Data were analysed thematically.

**Results:**

Most study rationales related to challenges facing the Indian health care system, rather than a wish to better understand phenomena related to teaching and learning. Similarly, most studies can be described as general evaluations of digital educational innovations, rather than educational research per se. They mostly explored questions related to student perception and intervention effectiveness, typically in the form of quantitative analysis of survey data or pre- and post-test results.

**Conclusions:**

The analysis revealed valuable insights into India-specific needs and challenges. The Indian health professions education system's size and unique challenges present opportunities for more nuanced, context-specific investigations and contributions to the wider digital education field. This, however, would require a broadening of methodological approaches, in particular rigorous qualitative designs, and a focus on addressing research-worthy educational phenomena.

**Supplementary Information:**

The online version contains supplementary material available at 10.1186/s12909-023-04552-2.

## Background

By 2030, the World Health Organization (WHO) has projected a global shortfall of 18 million health workers, primarily in low- and middle-income countries (LMICs) [1]. India is currently a major producer of health professionals in the world, but proportionally to the size of its population, the number of health professionals is inadequate and medical institutions in the country struggle with the poor quality of their personnel [2, 3]. The rise in non-communicable diseases during the past decades has put new pressure on the health system, and there is a great need for more and appropriately educated health professionals. Given the global shortage, this means that tertiary institutions must educate more health professionals and swiftly.

The Indian health professions education system is facing a number of challenges: there is a shortage of teaching staff, outdated didactic teaching styles, lack of faculty training, and logistical barriers when providing continuing education and training to health professionals in remote areas [3, 4]. A review by Frehywot et al. [5] suggests that digital education can help address some of these issues, e.g. by allowing for teachers to reach more students with engaging and interactive learning opportunities, regardless of their physical location. However, aside from the emergency remote teaching during the COVID-19 lockdowns, the Indian health professions education system has only limited experiences with digital education, and we know little about what approaches work best in Indian contexts which are distinctively different from those frequently described in health professional education journals [6].

To fully reap the benefits of digitalization, new educational initiatives should be accompanied by educational research that explores the effect and feasibility of different digital educational innovations in the Indian context. A good starting point is to assess the current, albeit limited, empirical research on digital health professions education in India. This will help us identify what is already known, and also suggest ways that future digital health professions education initiatives in India can profit from and contribute to the research on digital education. In addition, focussing on an educational environment that is infrequently discussed, we may also illuminate global challenges and benefits.

### Objective

This paper discusses strengths, weaknesses, thematic gaps, and future research opportunities with respect to Indian research in digital health professions education in order to understand how the digital might address the global shortage of health professionals. We conduct a scoping review of published empirical research related to digital health professions education in India. The objective is to synthesise the rationales, objectives, designs, methodologies, and contributions of the studies, highlighting aspects that are relevant to understanding the Indian context.

## Methods

A scoping review is a form of literature review that may be used to assess the nature and extend of research in a given field [7]. This is appropriate for our purposes, as we are not primarily synthesizing findings, but rather scoping a body of literature in order to understand what questions have been asked, and how they have been addressed [8]. To identify relevant publications for inclusion in the review we searched four research databases (Web of Science, PubMed, EBSCO Education Research Complete, and PsycInfo). This was done with a search string consisting of three elements, namely terms related to digital education (*n* = 174), terms related to health professions (*n* = 30), and terms related to India and Indian states (*n* = 43). Only English search terms were used. The search string and all search terms are included in the online supplementary materials (Appendix 1). At the point of searching the databases (July 2022), we did not limit the search to any specific period. We used the online review tool Covidence to manage the screening and data extraction processes.

**Fig. 1:**
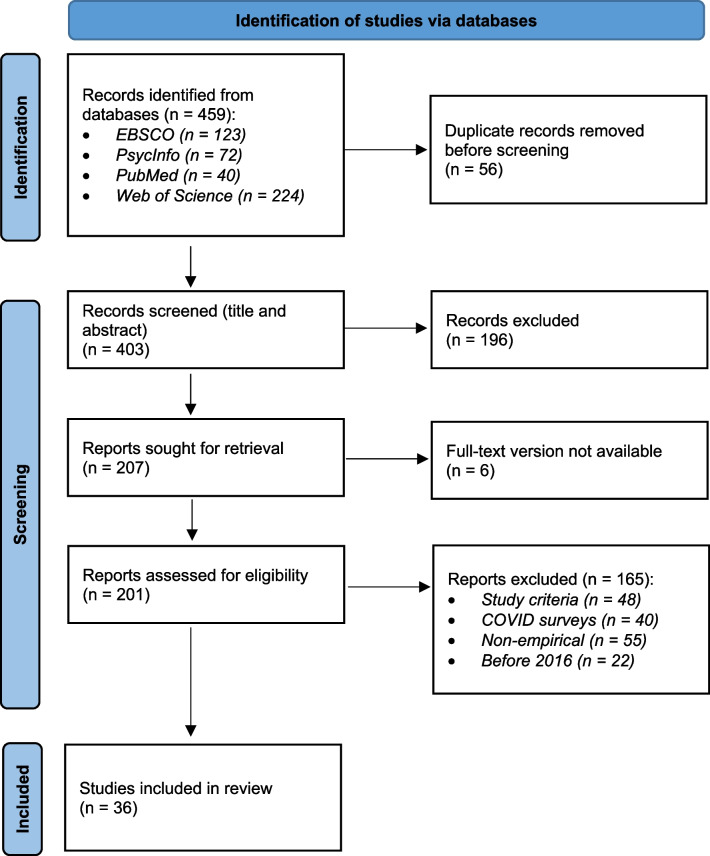
PRISMA flow diagram

We screened the titles and abstracts, then full texts, to determine whether they were suitable for inclusion in the review. Articles were included if they reported on empirical studies into digital educational innovations in the formal health professions education system of India. By *empirical research* we mean qualitative, quantitative, or mixed methods research analysing new or pre-existing datasets, e.g. from observations or experiments. We define *digital educational innovations* as any form of teaching and learning through digital platforms with digital tools and media. With the *formal health professions education system* we refer to the public and private institutions, who are responsible for providing accredited education and training to health professionals in India. In order to support our main goal of addressing the current global health crises, we excluded COVID lockdown surveys. We also excluded articles where we could not obtain the full texts. Furthermore, studies published before 2016 were excluded to retain the focus on contemporary digital technologies. This screening process consisted of two phases, both based on the mentioned criteria. First, we screened only titles and abstract, which led to an exclusion of 196 articles. This was followed by an in-depth screening of the full texts, leading to a further 165 articles being excluded. See Fig. 1 for a PRISMA flow diagram [9].

The screening process led us to a final pool of 36 included studies. From each study, we extracted data about study rationale, research design and methodology, study participants, study objective, and contributions. The data were analysed using a thematic approach to identify patterns, similarities, and differences among the studies. This means that we did not employ an a priori framework, but instead derived sub-themes (e.g. types of study objectives) inductively [10]. In line with Arksey & O’Malley [7], we did not do a formal assessment of study quality, but will make comment on quality throughout the paper.

## Results

**Fig. 2:**
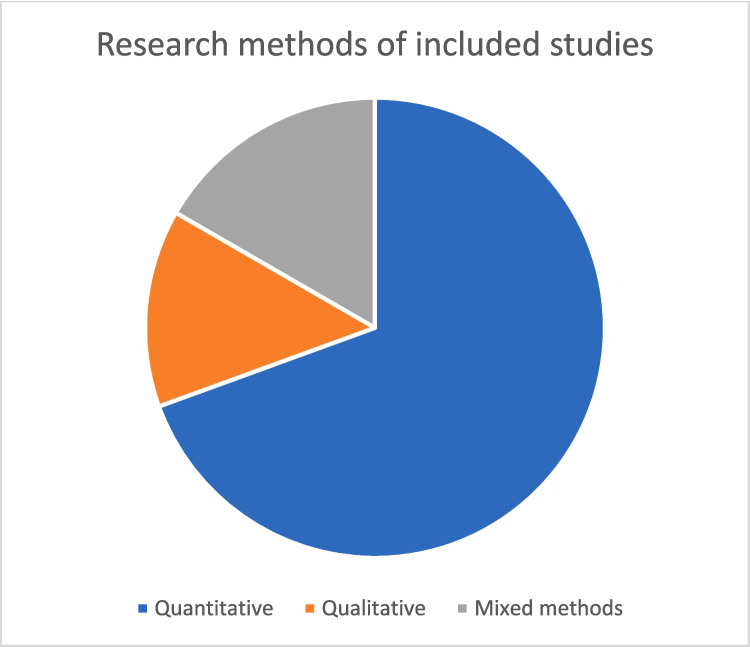
Research methods of the included studies

Of the 36 studies, 25 used only quantitative methods and five used only qualitative methods. The remaining six studies employed mixed methods, usually with the quantitative part as the most prominent (see Fig. 2). Almost half of the studies were observational (17). The remaining studies used various experimental designs, most often as part of interventional studies attempting to measure the effect of some digital educational innovation.

Most of the studies did not address a gap or phenomenon from the broader digital education literature. Instead, they took the form of more general evaluations of a teaching format or course design that the authors were using in their own role as teachers or trainers. The examined teaching–learning formats included both blended and hybrid models as well as different forms of fully online distance education. The digital tools at the focus of these formats included instant messengers, mobile phones, learning management systems, online quizzes, and open educational resources. Further details about each study are included in the online supplementary materials (Appendix 2).

### Target groups

Included studies addressed both formal health professions education, from e.g. medical or nursing colleges, as well as continuing education and other in-service training targeting health professionals. Seventeen papers study undergraduate (bachelor’s) level students, five study postgraduate (master’s) level students, 16 papers study in-service health professionals, and five study teaching staff.

The disciplines and levels targeted are shown with frequencies in Table 1. Following Indian tradition, nursing, midwifery, and *auxiliary nurse midwives* (ANM) are grouped together. Community health workers (CHW) and accredited social health activists (ASHA) are all *in-service*.

**Table 1:** Number of studies targeting each health professions group. Some studies target several groups

	**Undergraduate**	**Postgraduate**	**In-service**	**Teachers**	**All levels**
Medicine	11	2	7	3	19
Nursing, midwifery, ANM	4	1	8	2	12
CHW/ASHA	-	-	6	0	7
Dentistry	2	1	2	0	5
Public health	0	1	2	1	4
Allied health	0	0	2	0	2
AYUSH	0	0	0	0	0
Pharmacy	0	0	0	0	0
All disciplines	17	5	16	5	

### Study rationales

The authors of the 36 included studies provided a number of different motivations for doing the research. Most often, these were not traditional study rationales, focussed on a knowledge gap, but rather contextual arguments for the introduction of digital education as a practical innovation. In these cases, the rationale for doing the research was simply to evaluate or document the innovation.

These innovation rationales are interesting because they highlight concerns that might be addressed by digital education in the Indian context. Among the most frequently cited are the introduction of a new national curriculum [11, 12], the need to reach remote areas [13, 14], and the campus lockdowns imposed during the COVID pandemic [15–18]. Other rationales for exploring digital educational practices include catering to linguistic and cultural diversity [19], alleviating the general scarcity of health professionals [14], and cutting costs [20]. A number of studies also highlighted disease specific training needs for health professionals in the Indian or local context. Those include skills related to cancer screening [21], mental health [22], oral cancer [23], global health [24], nutrition [25], anatomy [26], maternal neonatal care [27], as well as care for mechanical ventilation patients [28]. The urgency to provide these skills to the health workforce was then listed as the motivation for a digital educational innovation.

The comparatively fewer studies with more traditional study rationales were mostly motivated by a wish to better understand certain challenges related to teaching and learning online. Among these are issues such as low attrition rates ([29] b), study group cohesion [30], use of inaccurate online resources [31], perception of digital education [32–35], as well as issues related to creating and running online courses [17, 36, 37].

### Study objectives

An analysis of the study objectives showed that the included studies have a great diversity in terms of what research questions they address. They can be broadly grouped into three types:

1. Study participants’ perception of an educational innovation
2. Effectiveness of an educational innovation
3. Experiences or behaviours of study participants

Nine studies had composite objectives that included both effectiveness and perception. Only U. Joshi et al. [36], who examined the cost of online course creation, did not include a study objective of either type.

#### Perceptions of an educational innovation

Twenty-three papers included measurements of study participant perception in the study objective, the most frequent type of study objective. Perception was often framed in terms of student opinion, acceptability, or attitude. Examples include perception of mobile learning [32, 33], perception of online formative assessment [38], and perception of blended learning [39]. A few studies examined perception as part of a qualitative analysis (e.g. [22]).

#### Effectiveness of an educational innovation

The studies that go beyond merely asking students about their opinion usually take the form of quantitative efficacy or feasibility studies. This was the case for 16 studies. These study objectives are typically found in the studies using pre- and post-test data collection as described above. In most cases, these studies seek to measure the effectiveness of different digital approaches, such as flipped classroom [11] or video-based online training [18].

#### Experiences and behaviours

Only five studies examined the experiences or behaviours of study participants. Two of these examined medical undergraduates’ use of electronic resources during their studies [12, 40], one explored the digital competencies of teaching staff [41], another examined experiences from creation of a Massive Open Online Course (MOOC) [37], and the fifth study explored retention and attrition in an online course for in-service health professionals [29]. The data types that were used to examine experiences and behaviours were more diverse than for perception and effectiveness, ranging from surveys and systems data, to interviews, observations, and document analysis.

### Types of data

**Fig. 3:**
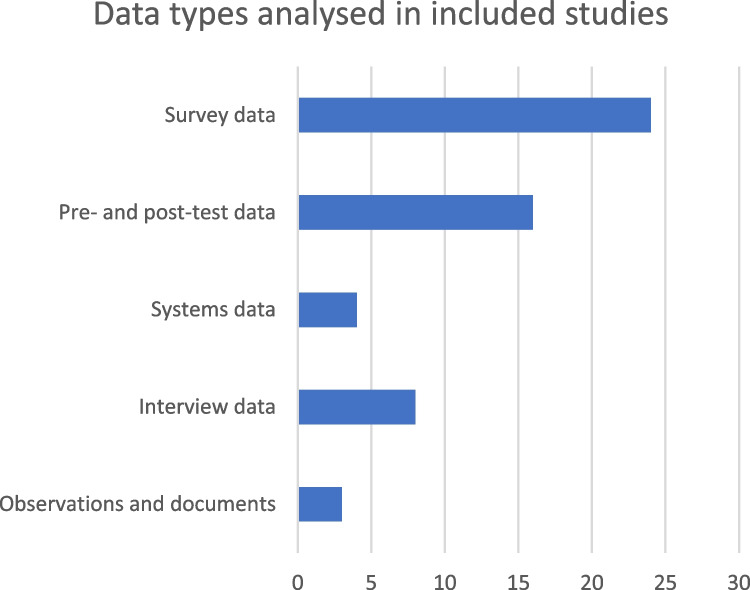
Frequency of different data types in the included studies

Following from the primarily quantitative body of literature, the collected data were mostly highly structured and suitable for statistical analysis. Below is a brief description of the different data types used in the studies, with their frequencies illustrated in Fig. 3.

#### Survey data

Data collected with surveys/questionnaires were the most common data type. This form was used in 24 of the 36 studies. Survey data were most often analysed quantitatively, although a few studies included (thematic) analyses of open-ended responses (e.g. [19, 30, 38]).

#### Pre- and post-test data

The second most common data type in the included studies was the use of pre- and post-test results of study participants. It was used in 16 studies. This form of data was collected in studies that sought to examine if a certain educational innovation was effective. Almost every experimental study used this form of data. The vast majority of the pre- and post-test studies did not include a control group, nor did they use statistical techniques to account for differences or potential confounding. Instead, intervention effectiveness was measured simply by comparing the results before and after training (e.g. [16, 28]).

#### Systems data

Systems data include automatically generated data about study participant behaviour in digital environments, e.g. number of logins to a learning management system or number of posts in an instant messenger chat. Only four of the included studies used systems data in their analyses, and it was most often as support for a primarily survey-based study (e.g. [19, 29]).

#### Interview data

Eight studies collected data with individual interviews (5) and focus groups (3). Of the five studies conducting individual interviews, three were with teachers/trainers [24, 27, 41]. Interview data were primarily used in qualitative analysis.

#### Observational data and document analysis

Three studies included other forms of unstructured data for qualitative analysis. One study included a round of prototype testing during which study participants were observed [20], while other studies included online observation and the collection of digital documents, e.g. in the form of teacher emails and submitted assignments [37].

### Contributions to the research literature

Twenty-three of the 36 studies can be described as general evaluations of a course or educational project. Typically, their study objectives were of the *perception* or *effectiveness* types described above. When measuring perception, they generally find that the study participants (learners) are positive about the new digital learning experience. In the few studies that go through the effort of comparing the effectiveness of two different practices, there are usually no significant difference (e.g. [34]). While these evaluation studies are undoubtedly valuable for the projects they evaluate, their contribution to knowledge beyond the case is limited.

**Fig. 4:**
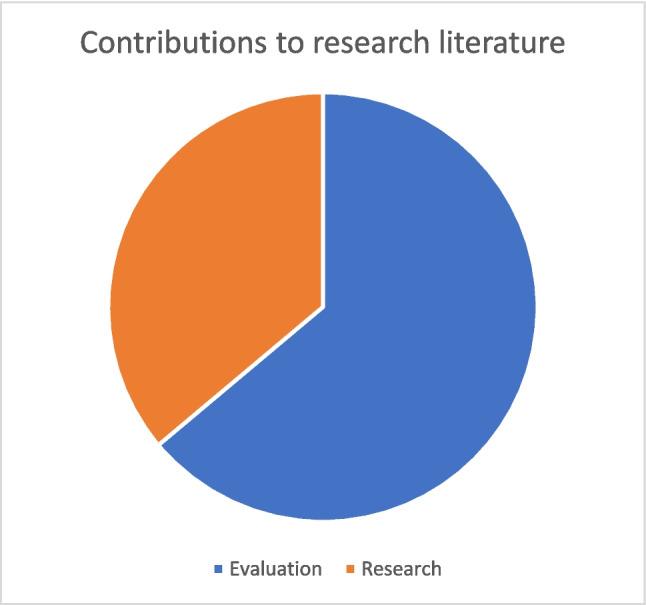
Type of contribution of included studies. Evaluations primarily contribute to the understanding of an intervention, while research primarily contributes to the research literature

The contributions to the broader digital education research literature are primarily found in the remaining 13 studies (see Fig. 4). Rather than presenting a project evaluation, these studies were intended to address a specific gap in our understanding of an educational phenomenon.

These phenomena were typically associated with specific *teaching practices* (6), such as peer teaching or problem-based learning, or *learner characteristics* (5), such as student retention or readiness for self-directed learning. The research topics and findings of these 13 studies are presented in Table 2.

**Table 2:** Research topics and study findings

Research topics	Findings
Self-Directed Learning Behaviour [42]	E-learning activity influenced Self-Directed Learning readiness among study participants. The activity was well perceived
Retention in online programmes [29]	The main reasons for quitting the course were high workload in the health facilities (75%) and poor internet connectivity (12%). A dedicated timeslot should be allotted for e-learning
Fostering group cohesion in online learning environments [30]	Including game-based activities can foster group cohesion. Around 90% of students agreed that game-based learning activities should be included as a regular part of online learning activities
Cost of online course creation [36]	Over a 10-month developmental period, the total costs were 208,814 USD, comprising expenses for human resources (61%), information technologies (33%), and infrastructure-related costs (6%)
Interactive virtual modules in simulation training [27]	Nurse Mentor Supervisors found the interactive virtual module to be engaging, innovative, relatable, and useful in teaching tips and tricks for simulation training. They thought the platform was largely accessible with some concerns around internet connectivity and devices
Reciprocal Peer Teaching and Flipped Classroom [17]	Mean knowledge and skills test scores improved significantly. Students agreed that this type of online learning provided good understanding, at a comfortable pace, with opportunity for interaction
Online formative assessment [38]	Most study participants agreed that the online quiz was a valuable learning activity and has potential to replace the face‑to‑face assessment. The online quizzes provided them feedback of classroom learning, helped in identifying the weak areas, and motivated them to study. The qualitative data showed that students like to receive a greater number of questions and are open to participating in quizzes in their spare time
Use of educational resources [12]	Traditional non-digital learning tools and practices are still popular among the first-year medical students. Along with these, students watch online tutorial videos and use smartphone applications for learning basic medical sciences. Medical teachers may use a combination of both to make teaching more acceptable to the students
Using WhatsApp for problem-based learning [43]	There were no significant differences in the post-test scores of the two groups. Except for enjoyment and interactiveness, there were no significant differences between student perception in the two groups. The students significantly preferred a combination of both types to either of them in isolation
Readiness toward online and blended learning [39]	A survey of entry level nursing students found that 54% of respondents felt blended learning would have positive effect on their learning and 70% of the respondents were ready to adopt blended learning
Teacher digital competencies and readiness for online teaching [41]	An analysis of interview data found that the faculty have acquired essential competencies for managing online education and most were positive about the experience with teaching online during lockdown
Interactive teaching in online courses [44]	When comparing the use of interactive teaching methods in online versus offline courses, this study found that the two groups benefited equally from the approach
Internet usage and online learning behaviour [40]	Medical students increasingly use the internet to access medical information, but this has not translated into increased participation in open online education

All of these 13 studies are based on empirical data from Indian health professions education, and as such provide insights into Indian contexts. While some of them may be considered specifically pertinent to Indian challenges and agendas, their focus on the educational phenomenon means that they can also contribute to the relevant international literatures.

## Discussion

Our analyses of the rationales, objectives, and contributions of the included studies present some insights into the particularities of the Indian context. Because of their close alignment with actual challenges from the Indian setting, the studies provide a valuable overview of both the contextual issues that the authors have tried to address with digital educational interventions, as well as the problems that emerged out of these interventions. However, it would be misleading to think of the Indian context as something uniform. Study environments, institutional infrastructure, and access to resources vary tremendously between regions. This is also illustrated by the fact that the Indian health professions education system is grappling with challenges that are prevalent across both low- and high-income countries. The identification of study rationales is perhaps the most valuable part of this scoping review as these function as contextual commentaries that can provide insight into India-specific needs in all their diversity, and thus contribute to the development of specific research agendas, as we outline below.

Mainstream medical education research, published in leading international journals, is dominated by studies from high income countries [6, 45]. This bias represents both a gap in the international evidence base and an important opportunity for Indian researchers. Based on this scoping review, we can identify ways in which rigorous empirical research from India would provide important new perspectives.

### Broadening of methodological approaches

Although many different research designs were employed in the 36 studies, the review reveals that most studies are based on perception/attitude surveys and attempts to measure student learning with pre- and post-test designs. This may well not be a problem that is exclusive to India: there have long been complaints about ‘the research we should be doing’ [46], which have persisted across much of the educational literature. From a research design perspective, a weakness is the lack of an appropriate control group. Using a single-arm design, they simply interpret any improvement in test score between the pre- and post-test as an argument for the effectiveness of the intervention. However, it is well established in educational research that *all interventions have a positive effect on learning*, and therefore a novel teaching practice should not be measured against “no teaching”, but rather against other (traditional) teaching practices [47]. Additionally, there is a longstanding concern that research into technologies does not distinguish between the pedagogy but merely focuses on comparing the modality [48]. Thus, in this call for further research into digital education beyond high-resource countries, it is worth reiterating the challenges experienced across many years of research, in order to avoid them. In particular, measuring increased student learning associated with a certain intervention is notoriously complex, especially in educational contexts that emphasize complex competencies over memorizing facts. Sometimes such measures are straightforward: linked to psychomotor skills that are readily measurable as in forms of simulation research. However, for more general changes to thinking or practice, it is unlikely that any form of short intervention (few days to a few weeks) will have an effect that is significantly different from that of a control group exposed to traditional practices. For the positive effects of an intervention to make a measurable difference to student learning, a much longer intervention is needed.

We note the near absence of learning analytics in the reviewed studies. Learning analytics, which are generated automatically by students as they use different digital tools and learning management systems, is widely used in international research on digital education [49, 50]. Although analytics are by no means a panacea, this represents an interesting possibility for future quantitative research projects, which rely less on surveys and pre- and post-test data.

As described in the results, the current research is almost exclusively quantitative and based in an implicit post-positivist paradigm that focuses on measurement of learning. This is again, not exclusive to India; it may be in part due to those with health sciences and/or digital backgrounds seeing *educational* research as an extension of their training. A first step may be a more frequent use of in-depth interviews and observation to supplement the more superficial qualitative analysis of open-ended responses to online surveys. Within educational research, qualitative research can provide another form of rich and detailed knowledge, that provides insights into the experiences and behaviours of students, teachers, and health professionals [51]. In particular, this type of data can *qualify* how the Indian context intersects with what is already known about digital learning.

### Where to for Indian research into digital health professions education?

Considering the enormity of the Indian health professions education system, 36 papers are an exceedingly small investigation. The analysis showed that the current body of research is primarily evaluative case studies. While valuable for the projects they evaluate, they often do not address a broader phenomenon or problem. *Peer learning*, *feedback and assessment*, *interprofessional education* and *social learning* designs are examples of educational approaches that could drive empirical research in an Indian setting.

This leaves two obvious opportunities for research endeavours. The first is to consider how digital education can or might unfold in India, *across contexts*, managing challenges or opportunities that operate beyond a single experience. The second is to contribute new knowledge to the general field of digital education research through adopting study objectives with a narrower focus, i.e. not evaluating an entire course, but singling out a research-worthy educational phenomenon that can be addressed in both observational and experimental study designs. An obvious place to start is by building study rationales which consider how the digital might intersect with challenges to Indian health professional education identified in this review, including remote healthcare contexts and training; linguistic and cultural diversity; and training in understaffed clinical environments.

Such moves are not a wholesale shift from the topics explored by the collected literature; rather we suggest reframing the problems to look beyond the immediate and the practical to providing insights into digital education as a field. Research which offers more abstract insights, can therefore have more long-standing impacts than what educators need to ‘do’ in an immediate local context. To give a concrete example, Anand et al. [19] explore mobile phone based learning and preterm care in a very concrete way. However, this study also offers the opportunity to study a broader phenomenon regarding how to teach touch-based care using mobile technologies. This is an area of great interest and generally under-researched [52]. A qualitative study which explored some of the tensions between pre-term care and phone learning might be very beneficial and might both provide insight into the broad issue of mobile learning in clinical care as well as the specific contextual requirements of Indian clinical environments.

Another important topic, and one where Indian educational research can impact practices and contribute to the international literature, is the use and sharing of open educational resources. Indian students already seek out and use such learning resources to support their own learning (e.g. [12]. The harmonized curricula and the large scale of the Indian health professions education system represents a unique context for research that explores best practice and barriers in the development, sharing, and use of digital educational resources and materials.

Recent years have seen many accrediting bodies within Indian health professions education develop their own guidelines for digital teaching and learning. During the COVID-19 campus lockdowns, the National Medical Commission, which is responsible for the medical curricula in all of India, published a report with guidelines for online learning and assessment [53]. Generally optimistic about the affordances of educational technology, these guidelines provide sound advice on best practices and common challenges. Aside from presenting the level of quality and student-centeredness that should be expected of digital health professions education in India, the guidelines also link the proposed practices to concepts and agendas from the educational research literature, such as formative assessment, plagiarism, student diversity, or active learning. This is valuable for educational researchers, who wish to move beyond evaluations and address a larger agenda.

### Strengths and limitations

This review is a diverse collaboration from a team of Indian and non-Indian authors. While this particular paper is not led by an Indian author, other papers from this project are. Although we believe this work to be rigorous and contribute valuable insights, there are limitations to our approach, most importantly the lack of dual independent screening and data extraction. Researcher triangulation might have increased validity and reduced the risk of bias in these processes. The narrow focus on Indian health professions education when identifying articles can also be seen as a limitation. A broader scope, for instance by exploring all of Indian higher education, or by expanding the search to more countries in the region, might have yielded further studies from stronger research traditions, which could be valuable inspiration for Indian health professions education researchers and others who are active in this field.

## Conclusions

This scoping review identifies an impetus towards understanding how digital education unfolds within Indian contexts. It also presents a number of opportunities for educational research in the Indian health professional context. This includes moving away from perception/attitude surveys towards more diverse data types such as learning analytics, qualitative interviews, and observations in- and outside the digital environments. A comprehensive research agenda along these lines would have the dual purpose of strengthening capacity at Indian health professions education institutions, while at the same time contributing a valuable Indian perspective to the international literature.

### Supplementary Information

**Additional file 1:**
**Appendix 1.** Search terms. **Appendix 2.** Included studies. 

## Data Availability

Data extracted for this review article are included in this published article and its supplementary information files.

## Abbreviations

ASHA: Accredited social health activist
ANM: Auxiliary nurse midwives
CHW: Community health worker
LMIC: Low- and middle-income country
MOOC: Massive open online course
WHO: World Health Organization
