# Supplementary material for: Digital education for health professionals in India: a scoping review of the research
Source: BMC Med Educ. 2023 Aug 9;23:561. doi: 10.1186/s12909-023-04552-2 (PMC10410828; doi:10.1186/s12909-023-04552-2)
Supplement: Supplementary file 1 — Additional file 1: Appendix 1. Search terms. Appendix 2. Included studies. [file 12909_2023_4552_MOESM1_ESM.docx]

Appendix 1 – Search terms

Structure of search string: *(Digital education terms) AND (Health professions terms) AND (Indian geographical terms)*

**Digital education terms**

("digital education*" OR "digital learn*" OR "digital instruction*" OR "digital self-learn*" OR "digital self learn*" OR "digital teaching" OR "digital course*" OR "digital training" OR "digital tool*" OR "digital library" OR "online learn*" OR "online instruction*" OR "online course*" OR "online training" OR "online education*" OR "online teaching" OR "e-instruction*" OR "e instruction*" OR "e-learn*" OR "e learn*" OR "e-teaching" OR "e teaching" OR "e-resources" OR "e resources" OR "blended learn*"OR "blended instruction*" OR "blended course*" OR "blended training" OR "blended education*" OR "blended teaching" OR "distributed learn*" OR "distributed instruction*" OR "distributed course*" OR "distributed training" OR "distributed education*" OR "distributed teaching" OR "mediated learn*" OR "mediated instruction*" OR "mediated course*" OR "mediated training" OR "mediated education*" OR "mediated teaching" OR "distance education*" OR "distance instruction* OR "distance learn*" OR "distance course*" OR "distance teaching" OR "distance training" OR "virtual education*" OR "virtual classroom" OR "virtual education*" OR "virtual learn*" OR "virtual instruction*" OR "virtual course*" OR "virtual training" OR "virtual teaching" OR "remote learn*" OR "remote instruction*" OR "remote course*" OR "remote training" OR "remote education*" OR "remote teaching" OR "multimedia learn*" OR "multimedia course*" OR "multimedia instruction*" OR "multimedia training" OR "multimedia education*" OR "multimedia teaching" OR "reusable learning object*" OR "web-based learn*" OR "web-based instruction*" OR "web-based teaching" OR "web-based course*" OR "web-based training" OR "web-based education*" OR "web based learn*" OR "web based instruction*" OR "web based teaching" OR "web based course*" OR "web based training" OR "web based education*" OR "internet-based learn*" OR "internet-based instruction*" OR "internet-based teaching" OR "internet-based course*" OR "internet-based training" OR "internet-based education" OR "internet based learn*" OR "internet based instruction*" OR "internet based teaching" OR "internet based course*" OR "internet based training" OR "internet based education*" OR "digitally mediated learn*" OR "digitally mediated instruction*" OR "digitally mediated course*" OR "digitally mediated training" OR "digitally mediated education*" OR "digitally mediated teaching" OR "simulation-based learn*" OR "simulation-based instruction*" OR "simulation-based education*" OR "simulation-based teaching" OR "simulation-based training" OR "simulation-based course*" OR "simulation based learn*" OR "simulation based instruction*" OR "simulation based education*" OR "simulation based teaching" OR "simulation based training" OR "simulation based course*" OR "computer-assisted learn*" OR "computer-assisted instruction*" OR "computer-assisted course*" OR "computer-assisted training" OR "computer-assisted education*" OR "computer-assisted teaching OR "computer assisted learn*" OR "computer assisted instruction*" OR "computer assisted course*" OR "computer assisted training" OR "computer assisted education*" OR "computer assisted teaching" OR "computer-based learn*" OR "computer-based instruction*"OR "computer-based course*" OR "computer-based training" OR "computer-based education*" OR "computer-based teaching OR "computer based learn*" OR "computer based instruction*" OR "computer based course*" OR "computer based training" OR "computer based education*" OR "computer based teaching" OR "computer-aided instruction" OR "computer-aided learn*" OR "computer-aided instruction*"OR "computer-aided course*" OR "computer-aided training" OR "computer-aided education*" OR "computer-aided teaching OR "computer aided learn*" OR "computer aided instruction*" OR "computer aided course*" OR "computer aided training" OR "computer aided education*" OR "computer aided teaching" OR "mobile learn*" OR "mobile instruction*" OR "mobile course*" OR "mobile training" OR "mobile education*" OR "mobile teaching" OR "video-based learn*" OR "video-based instruction*" OR "video-based course*" OR "video-based training" OR "video-based education*" OR "video-based teaching" OR "video based learn*" OR "video based instruction*" OR "video based course*" OR "video based training" OR "video based education*" OR "video based teaching" OR "video-teleconference" OR "tele-learn*" OR "tele-instruction*" OR "tele-course*" OR "tele-training" OR "tele-education*" OR "tele-teaching" OR "tele learn*" OR "tele instruction*" OR "tele course*" OR "tele training" OR "tele education*" OR "tele teaching" OR "audio response system" OR "learning management system" OR "course management system" OR "Massive Open Online Course*")

**Health professions terms**

(medicine OR mbbs OR md OR medical OR meded OR nursing OR nurse* OR dentist* OR "health professional*" OR "health professions education" OR "human resources for health" OR "healthcare workforce" OR "health worker*" OR "health workforce" OR "health education" OR "AYUSH" OR Ayurveda OR Yoga OR Unani OR Siddha OR Homeopathy OR ("Indian system" AND "Medicine") OR (Indigenous AND Medicine) OR "Indian Medicine and Homeopathy" OR "ISM & H" OR "ANM" OR "Auxiliary Nurses and Midwives" OR midwives OR midwifery OR allopathy)

**Indian geographical terms**

(India OR Indian OR "Andhra Pradesh" OR "Arunachal Pradesh" OR Assam OR Bihar OR Chhattisgarh OR Goa OR Gujarat OR Haryana OR "Himachal Pradesh" OR Jharkhand OR Karnataka OR Kerala OR "Madhya Pradesh" OR Maharashtra OR Manipur OR Meghalaya OR Mizoram OR Nagaland OR Odisha OR Orissa OR Punjab OR Rajasthan OR Sikkim OR "Tamil Nadu" OR Telangana OR Tripura OR "Uttar Pradesh" OR Uttarakhand OR "West Bengal" OR Andaman "Nicobar Islands" OR Chandigarh OR Dadra OR "Nagar Haveli" OR Daman OR Diu OR Delhi OR Jammu OR Kashmir OR Ladakh OR Lakshadweep OR Puducherry)

Appendix 2 – Included studies

| **Author(s)** | **Title** | **Methods** | **Data** | **Disciplines** | **Levels** | **Objective type** | **Study focus** |
| --- | --- | --- | --- | --- | --- | --- | --- |
| Anand et al. (2021) | Dissemination of Best Practices in Preterm Care Through a Novel Mobile Phone-Based Interactive e-Learning Platform | Quantitative | Survey; LA and systems data | Nursing/Midwife/ANM | Undergraduate; In-service | Perception | Mobile learning |
| Aristotle et al. (2021) | Effectiveness of flipped classroom model in teaching histology for first-year MBBS students based on competency-based blended learning: An interventional study | Mixed methods | Survey; Pre- and post-test | Medicine | Undergraduate | Perception; Effectiveness | Flipped classroom |
| Babu et al. (2021) | Leveraging Technology for Nation-Wide Training of Healthcare Professionals in Cancer Screening in India: a Methods Article | Quantitative | Survey | Medicine; Nursing/Midwife/ANM; Dentistry; Allied Health; Public Health; Community Health Worker/ASHA | In-service | Perception | General evaluation |
| Bairy et al. (2021) | Capacity Building in Mental Health for Bihar: Overview of the 1-Year Blended Training Program for Nonspecialist Medical Officers | Quantitative | Survey; Pre- and post-test | Medicine | In-service | Perception; Effectiveness | General evaluation |
| Balasubramaniam et al. (2018) | Blending virtual with conventional learning to improve student midwifery skills in India | Quantitative | Pre- and post-test | Nursing/Midwife/ANM | Undergraduate | Effectiveness | General evaluation |
| Bankar (2021) | Self Directed Learning Behaviour- Impact of E-Learning Activity on Students | Quantitative | Survey; Pre- and post-test | Medicine | Undergraduate | Perception | SDL readiness |
| Basu et al. (2020) | Attitude towards mobile learning among resident doctors involved in undergraduate medical education at a government medical college in Delhi, India. | Quantitative | Survey | Medicine | Graduate; In-service; Teaching staff | Perception | Mobile learning |
| Bhuvaneshwari et al. (2021) | Knowledge, attitude, and challenges in digital learning using smartphones among dental students of South India: a cross-sectional survey | Quantitative | Survey | Dentistry | Undergraduate; Graduate | Perception | Mobile learning |
| Chakravarty et al. (2016) | Blended Training for Frontline Health Functionaries: Is this the Way Ahead? | Quantitative | Pre- and post-test | Nursing/Midwife/ANM; Community Health Worker/ASHA | In-service | Effectiveness | Blended learning |
| Chauhan et al. (2019) | Asynchronous versus traditional teaching for MBBS undergraduate students-effectiveness and students perspectives - A pilot study | Quantitative | Survey; Pre- and post-test | Medicine | Undergraduate | Perception; Effectiveness | Online learning |
| Dash (2019) | Google classroom as a learning management system to teach biochemistry in a medical school | Quantitative | Survey | Medicine | Undergraduate | Perception; Effectiveness | Google Classroom |
| Dhanasekaran et al. (2020 a) | Capacity building of gynecologists in cancer screening through hybrid training approach | Quantitative | Pre- and post-test | Medicine | In-service | Effectiveness | Hybrid training |
| Dhanasekaran et al. (2020 b) | Factors influencing the retention of participants in online cancer screening training programs in India | Quantitative | Survey; LA and systems data | Medicine; Dentistry; Community Health Worker/ASHA | In-service | Behaviour /experiences | Retention |
| Elfaki (2022) | Undergraduate Nursing Students' Knowledge, Attitude: Care of Patients of Mechanical Ventilators | Quantitative | Survey; Pre- and post-test | Nursing/Midwife/ANM | Undergraduate | Perception; Effectiveness | General evaluation |
| Hirkani et al. (2022) | Strategies to foster group cohesion in online learning environments: use of crossword and Hybrid Medical Pictionary | Mixed methods | Survey | Medicine | Undergraduate | Perception | Group cohesion |
| Jadhav et al. (2022) | A Novel Online Dissection Course on Lower Limb Anatomy During the COVID-19 Pandemic | Quantitative | Survey | Medicine | Undergraduate | Perception | Online learning |
| U. Joshi et al. (2022) | Assessing costs of developing a digital program for training community health workers to deliver treatment for depression: A case study in rural India | Quantitative | Survey | Community Health Worker/ASHA | In-service | - | Cost of course development |
| P. Joshi et al. (2022) | Dissemination of Cardiopulmonary Resuscitation Training for Nurses Treating Coronavirus Disease-2019 Patients: A Single-arm Pre-experimental Study | Quantitative | Pre- and post-test | Nursing/Midwife/ANM | In-service | Effectiveness | General evaluation |
| Kalra et al. (2022) | Super Divya to the rescue! Exploring Nurse Mentor Supervisor perceptions on a digital tool to support learning and engagement for simulation educators in Bihar, India | Qualitative | Interviews (individual) | Nursing/Midwife/ANM; Other | In-service; Teaching staff | Perception | Interactive virtual module |
| Khapre et al. (2021) | Effectiveness of Integrated Google Classroom, Reciprocal Peer Teaching and Flipped Classroom on Learning Outcomes of Research Methodology: A Natural Experiment | Quantitative | Survey; Pre- and post-test | Public Health | Graduate | Perception; Effectiveness | Peer teaching; Flipped classroom |
| Kour (2019) | Usefulness of Teleconferencing for Nursing Distance Students in India. | Quantitative | Survey; Interviews (individual) | Nursing/Midwife/ANM | Graduate | Perception | Teleconferencing |
| Mondal et al. (2021 a) | Medical students' perception on the usefulness of online formative assessment: A single-center, mixed-method, pilot study | Mixed methods | Survey | Medicine | Undergraduate | Perception | Online formative assessment |
| Mondal et al. (2021 b) | Educational resources used by 1st-year medical students | Quantitative | Survey | Medicine | Undergraduate | Behaviour /experiences | Use of E-resources |
| Muke et al. (2019) | Acceptability and feasibility of digital technology for training community health workers to deliver brief psychological treatment for depression in rural India | Mixed methods | Survey; Interviews (group); LA and systems data; Pre- and post-test; Support calls from students during the course | Community Health Worker/ASHA | In-service | Perception; Effectiveness | General evaluation |
| Muke et al. (2020) | Digital Training for Non-Specialist Health Workers to Deliver a Brief Psychological Treatment for Depression in Primary Care in India: Findings from a Randomized Pilot Study | Qualitative | Interviews (group); Observations (f2f) | Community Health Worker/ASHA | In-service | Perception | General evaluation |
| Nagaraj et al. (2021) | Effectiveness of blended learning in radiological anatomy for first year undergraduate medical students | Quantitative | Pre- and post-test | Medicine | Undergraduate | Perception; Effectiveness | Blended learning |
| Nethan et al. (2020) | Project ECHO: A potential best-practice tool for training healthcare providers in oral cancer screening and tobacco cessation | Quantitative | Pre- and post-test | Medicine; Nursing/Midwife/ANM; Dentistry; Allied Health; Public Health | In-service; Health researchers | Effectiveness | General evaluation |
| Ponnaiah et al. (2022) | Design and implementation challenges of massive open online course on research methods for Indian medical postgraduates and teachers –descriptive analysis of inaugural cycle | Mixed methods | Survey; Observations (online); LA and systems data; Document review (emails, assignments) | Medicine; | Graduate; Teaching staff | Behaviour /experiences | General evaluation |
| Protsiv & Atkins (2016) | The experiences of lecturers in African, Asian and European universities in preparing and delivering blended health research methods courses: a qualitative study | Qualitative | Interviews (individual) | Public Health | Teaching staff | Perception | Blended learning |
| Scott et al. (2022) | '[We] learned how to speak with love': a qualitative exploration of accredited social health activist (ASHA) community health worker experiences of the Mobile Academy refresher training in Rajasthan, India. | Qualitative | Interviews (individual); Interviews (group) | Community Health Worker/ASHA | In-service | Perception | General evaluation |
| Sengupta & Sur (2021) | Effectiveness of WhatsApp as a Teaching Learning Tool for Problem Based Learning in Pharmacology: A Quasi-experimental Study | Quantitative | Survey; Pre- and post-test | Medicine | Undergraduate | Perception; Effectiveness | WhatsApp and PBL |
| Sharma et al. (2021) | Effectiveness of Video-Based Online Training for Health Care Workers to Prevent COVID-19 Infection: An Experience at a Tertiary Care Level Institute, Uttar sand, India | Quantitative | Survey; Pre- and post-test | Nursing/Midwife/ANM | In-service | Effectiveness | General evaluation |
| Swaminathan et al. (2021) | Entry level nursing graduate students' perception and readiness toward online component of blended learning: A mixed method study | Mixed methods | Survey; Interviews (group) | Nursing/Midwife/ANM | Undergraduate | Perception | Student readiness for OBL |
| Valsaraj et al. (2021) | Faculty experiences on emergency remote teaching during COVID-19: a multicentre qualitative analysis | Qualitative | Interviews (individual) | Medicine; Nursing/Midwife/ANM | In-service; Teaching staff | Behaviour /experiences | Teacher digital competencies and readiness |
| Veeraiyan et al. (2022) | Comparison of Interactive Teaching in Online and Offline Platforms among Dental Undergraduates | Quantitative | Pre- and post-test | Dentistry | Undergraduate | Effectiveness | Interactive teaching |
| Venkatesh et al. (2017) | A survey on internet usage and online learning behaviour among medical undergraduates | Quantitative | Survey | Medicine | Undergraduate | Behaviour /experiences | Use of E-resources |

References

Anand, P., Thukral, A., Deorari, A., National Neonatology Forum Network, Sethi, A., Kumar, A., Yadav, A., Balachandran, A., Raj, A., Kumar, A., Mallick, A. K., Mondal, B., Balachander, B., Saha, B., Varghese, B., M.S, C., Sajeev, C., Nanda, D., Thomas, D., … Ghosh, U. (2021). Dissemination of Best Practices in Preterm Care Through a Novel Mobile Phone-Based Interactive e-Learning Platform. *Indian Journal of Pediatrics*, *88*(11), 1068–1074. https://doi.org/10.1007/s12098-021-03689-6

Aristotle, S., Subramanian, S., & Jayakumar, S. (2021). Effectiveness of flipped classroom model in teaching histology for first-year MBBS students based on competency-based blended learning: An interventional study. *Journal of Education and Health Promotion*, *10*(1). https://doi.org/10.4103/jehp.jehp_467_20

Babu, R., Dhanasekaran, K., Mehrotra, R., & Hariprasad, R. (2021). Leveraging Technology for Nation-Wide Training of Healthcare Professionals in Cancer Screening in India: A Methods Article. *Journal of Cancer Education*, *36*(5), 950–956. https://doi.org/10.1007/s13187-020-01720-6

Bairy, B., Ganesh, A., Kaur, S., Chand, P., Kumar, C., Manjunatha, N., Math, S., Sinha, N., & Arora, S. (2021). Capacity Building in Mental Health for Bihar: Overview of the 1-Year Blended Training Program for Nonspecialist Medical Officers. *Journal of Neurosciences in Rural Practice*, *12*(02), 329–334. https://doi.org/10.1055/s-0041-1722842

Balasubramaniam, S. M., Bhargava, S., Agrawal, N., Asif, R., Chawngthu, L., Sinha, P., Kumar, S., & Sood, B. (2018). Blending virtual with conventional learning to improve student midwifery skills in India. *Nurse Education in Practice*, *28*, 163–167. https://doi.org/10.1016/j.nepr.2017.10.028

Bankar, M. A. (2021). Self Directed Learning Behaviour- Impact of E-Learning Activity on Students. *Journal of Clinical and Diagnostic Research*, *15*(1). https://doi.org/10.7860/JCDR/2021/46274.14419

Basu, S., Marimuthu, Y., Sharma, N., Sharma, P., Gangadharan, N., & Santra, S. (2020). Attitude towards mobile learning among resident doctors involved in undergraduate medical education at a government medical college in Delhi, India. *Journal of Education and Health Promotion*, *9*, 321. https://doi.org/10.4103/jehp.jehp_443_20

Bhuvaneshwari, G., Nitya, K., Karthikeyan, M., Purushotham, M., Vikram, S. A., & Kirubakaran, A. K. (2021). Knowledge, attitude, and challenges in digital learning using smartphones among dental students of South India: A cross-sectional survey. *Journal of International Oral Health*, *13*(2), 181–188. https://doi.org/10.4103/jioh.jioh_285_20

Chakravarty, N., Nallala, S., Mahapatra, S., Chaudhury, P., Sultana, F., & Bhattacharjee, S. (2016). Blended Training for Frontline Health Functionaries: Is this the Way Ahead? *International Journal of Preventive Medicine*, *7*, 37. https://doi.org/10.4103/2008-7802.176002

Chauhan, V. D., Kalra, J., Kalra, V., Negi, G., & Agarwal, P. (2019). Asynchronous versus traditional teaching for MBBS undergraduate students-effectiveness and students perspectives—A pilot study. *International Journal of Applied and Basic Medical Research*, *9*(2), 69–72. https://doi.org/10.4103/ijabmr.IJABMR_260_18

Dash, S. (2019). Google classroom as a learning management system to teach biochemistry in a medical school. *Biochemistry and Molecular Biology Education*, *47*(4), 404–407. https://doi.org/10.1002/bmb.21246

Dhanasekaran, K., Babu, R., Kumar, V., Singh, S., & Hariprasad, R. (2020). Factors influencing the retention of participants in online cancer screening training programs in India. *BMC Medical Education*, *20*(1), 220. https://doi.org/10.1186/s12909-020-02144-y

Elfaki, B. (2022). Undergraduate Nursing Students’ Knowledge, Attitude: Care of Patients of Mechanical Ventilators. *Journal of Algebraic Statistics*, *13*(2), 58–67.

Hirkani, M., Hegde, G., Kamath, R., Sonwane, T., Angane, E., & Gajbhiye, R. (2022). Strategies to foster group cohesion in online learning environments: Use of crossword and Hybrid Medical Pictionary. *Advances in Physiology Education*, *46*(1), 30–34. https://doi.org/10.1152/advan.00116.2021

Jadhav, S., Bharambe, V. K., Pathak, V. S., Khurjekar, A. P., Navandar, R. L., & Arunprasad, V. K. (2022). A Novel Online Dissection Course on Lower Limb Anatomy During the COVID-19 Pandemic. *Cureus Journal of Medical Science*, *14*(3), e23081. https://doi.org/10.7759/cureus.23081

Joshi, P., Das, S., Thomas, M., Mawar, S., Garg, R., Shariff, A., & Gopichandran, L. (2022). Dissemination of Cardiopulmonary Resuscitation Training for Nurses Treating Coronavirus Disease-2019 Patients: A Single-arm Pre-experimental Study. *Indian Journal of Critical Care Medicine*, *26*(3), 327–330. https://doi.org/10.5005/jp-journals-10071-24128

Joshi, U., Naslund, J., Anand, A., Tugnawat, D., Vishwakarma, R., Bhan, A., Patel, V., & Lu, C. (2022). Assessing costs of developing a digital program for training community health workers to deliver treatment for depression: A case study in rural India. *Psychiatry Research*, *307*. https://doi.org/10.1016/j.psychres.2021.114299

Kalra, A., Siju, M., Jenny, A., Spindler, H., Madriz, S., Baayd, J., Handu, S., Ghosh, R., Cohen, S., & Walker, D. (2022). Super Divya to the rescue! Exploring Nurse Mentor Supervisor perceptions on a digital tool to support learning and engagement for simulation educators in Bihar, India. *BMC Medical Education*, *22*(1), 206. https://doi.org/10.1186/s12909-022-03270-5

Khapre, M., Sinha, S., & Kaushal, P. (2021). Effectiveness of Integrated Google Classroom, Reciprocal Peer Teaching and Flipped Classroom on Learning Outcomes of Research Methodology: A Natural Experiment. *Cureus*, *13*(7), e16176. https://doi.org/10.7759/cureus.16176

Kour, J. J. (2019). Usefulness of Teleconferencing for Nursing Distance Students in India. *Asian Journal of Distance Education*, *14*(1), 77–87.

Mondal, H., Dutta, S., Mondal, S., Sahoo, M., Saha, K., & Mondal, S. (2021). Educational resources used by 1st-year medical students. *Journal of the Anatomical Society of India*, *70*(3), 130–135. https://doi.org/10.4103/JASI.JASI_16_20

Mondal, H., Sahoo, M., Samantaray, R., & Mondal, S. (2021). Medical students’ perception on the usefulness of online formative assessment: A single-center, mixed-method, pilot study. *Journal of Education and Health Promotion*, *10*(1). https://doi.org/10.4103/jehp.jehp_1198_20

Muke, S., Shrivastava, R., Mitchell, L., Khan, A., Murhar, V., Tugnawat, D., Shidhaye, R., Patel, V., & Naslund, J. (2019). Acceptability and feasibility of digital technology for training community health workers to deliver brief psychological treatment for depression in rural India. *Asian Journal of Psychiatry*, *45*, 99–106. https://doi.org/10.1016/j.ajp.2019.09.006

Muke, S., Tugnawat, D., Joshi, U., Anand, A., Khan, A., Shrivastava, R., Singh, A., Restivo, J., Bhan, A., Patel, V., & Naslund, J. (2020). Digital Training for Non-Specialist Health Workers to Deliver a Brief Psychological Treatment for Depression in Primary Care in India: Findings from a Randomized Pilot Study. *International Journal of Environmental Research and Public Health*, *17*(17). https://doi.org/10.3390/ijerph17176368

Nagaraj, C., Yadurappa, S. B., Anantharaman, L. T., Ravindranath, Y., & Shankar, N. (2021). Effectiveness of blended learning in radiological anatomy for first year undergraduate medical students. *Surgical and Radiologic Anatomy*, *43*(4), 489–496. https://doi.org/10.1007/s00276-020-02572-x

Nethan, S. T., Hariprasad, R., Babu, R., Kumar, V., Sharma, S., & Mehrotra, R. (2020). Project ECHO: A potential best-practice tool for training healthcare providers in oral cancer screening and tobacco cessation. *Journal of Cancer Education*, *35*(5), 965–971. https://doi.org/10.1007/s13187-019-01549-8

Ponnaiah, M., Bhatnagar, T., Ganeshkumar, P., Bhar, D., Elumalai, R., Vijayageetha, M., Abdulkader, R. S., Chaudhuri, S., Sharma, U., & Murhekar, M. V. (2022). “Design and implementation challenges of massive open online course on research methods for Indian medical postgraduates and teachers –descriptive analysis of inaugural cycle”. *BMC Medical Education*, *22*(1), 369. https://doi.org/10.1186/s12909-022-03423-6

Protsiv, M., & Atkins, S. (2016). The experiences of lecturers in African, Asian and European universities in preparing and delivering blended health research methods courses: A qualitative study. *Global Health Action*, *9*, 28149. https://doi.org/10.3402/gha.v9.28149

Scott, K., Ummer, O., Chamberlain, S., Sharma, M., Gharai, D., Mishra, B., Choudhury, N., & LeFevre, A. E. (2022). ‘[We] learned how to speak with love’: A qualitative exploration of accredited social health activist (ASHA) community health worker experiences of the Mobile Academy refresher training in Rajasthan, India. *BMJ Open*, *12*(6), e050363. https://doi.org/10.1136/bmjopen-2021-050363

Sengupta, P., & Sur, T. (2021). Effectiveness of WhatsApp as a Teaching Learning Tool for Problem Based Learning in Pharmacology: A Quasi-experimental Study. *Journal of Clinical and Diagnostic Research*, *15*(10), JC5–JC9. https://doi.org/10.7860/JCDR/2021/51256.15568

Sharma, R., Mohanty, A., Singh, V., Vishwas, A., Gupta, P., Jelly, P., Gupta, P., & Rao, S. (2021). Effectiveness of Video-Based Online Training for Health Care Workers to Prevent COVID-19 Infection: An Experience at a Tertiary Care Level Institute, Uttar sand, India. *Cureus Journal of Medical Science*, *13*(5). https://doi.org/10.7759/cureus.14785

Swaminathan, N., Ravichandran, L., Ramachandran, S., Milanese, S., Singaravelu, R., & Govindaraj, P. (2021). Entry level nursing graduate students’ perception and readiness toward online component of blended learning: A mixed method study. *Journal of Education and Health Promotion*, *10*(1). https://doi.org/10.4103/jehp.jehp_771_20

Valsaraj, B. P., More, B., Biju, S., Payini, V., & Pallath, V. (2021). Faculty experiences on emergency remote teaching during COVID-19: A multicentre qualitative analysis. *Interactive Technology and Smart Education*, *18*(3), 319–344. https://doi.org/10.1108/ITSE-09-2020-0198

Veeraiyan, D. N., Varghese, S. S., Rajasekar, A., Karobari, M. I., Thangavelu, L., Marya, A., Messina, P., & Scardina, G. A. (2022). Comparison of Interactive Teaching in Online and Offline Platforms among Dental Undergraduates. *International Journal of Environmental Research and Public Health*, *19*(6), 3170. https://doi.org/10.3390/ijerph19063170

Venkatesh, S., Chandrasekaran, V., Dhandapany, G., Palanisamy, S., & Sadagopan, S. (2017). A survey on internet usage and online learning behaviour among medical undergraduates. *Postgraduate Medical Journal*, *93*(1099), 275–279. https://doi.org/10.1136/postgradmedj-2016-134164
